# Long-Acting HIV Treatment and Prevention: Closer to the Threshold

**DOI:** 10.9745/GHSP-D-17-00206

**Published:** 2017-06-27

**Authors:** Matthew Barnhart

**Affiliations:** a Associate Editor, *Global Health: Science and Practice*, and Medical Officer, U.S. Agency for International Development, Washington, DC, USA.

## Abstract

Substantial progress has been made toward viable, practical long-acting approaches to deliver HIV treatment and prevention through: (1) continued improvements in long-acting antiretrovirals (ARVs); (2) better innovative delivery systems; and (3) collaboration of willing partners to advance new ARVs. More progress on those 3 fronts is still needed to arrive at the goal of optimized HIV treatment and prevention for all who would benefit—and of finally controlling the HIV epidemic.

## WHY WE NEED LONG-ACTING ANTIRETROVIRALS

With 18.2 million people currently receiving HIV treatment, 2.1 million new HIV infections per year, and guidelines recommending that treatment be offered to all 36.7 million people living with HIV,^1^ the need to improve HIV treatment and prevention is clear. Long-acting antiretrovirals (ARVs) are one approach that holds great promise to enable major gains in efficiency and effectiveness.

The crucial advantage of long-acting ARVs stems from their potential to improve patients' adherence, which is critical for good outcomes both for treatment and prevention. For treatment, being able to directly administer a long-acting regimen on a monthly or less frequent basis to a patient might minimize risk of treatment failure and resistance due to inconsistent adherence while also potentially reducing the need for costly laboratory tests to monitor treatment efficacy. For prevention, the benefits of long-acting agents compared with daily oral agents may be even more compelling, as oral pre-exposure prophylaxis (PrEP) has been demonstrated to be very effective when used,^2^^,^^3^ but poor adherence has been reported in several studies,^4^^,^^5^ limiting impact. In addition to the greater efficacy that long-acting ARVs might bring, they may also have potential to reduce drug costs, since long-acting formulations typically contain agents that are effective at a very low dosage, which, other things equal, can translate into lower manufacturing costs.

Long-acting ARVs have the potential to improve patients' adherence, which is critical for good outcomes both for treatment and prevention.

New interventions should ideally be highly effective, safe, user-friendly, of suitable duration, inexpensive, socially acceptable, and easy to implement.^6^ Several long-acting candidates for HIV prevention and treatment that may meet these criteria are now advancing closer to the threshold for global health impact.

Several long-acting ARV candidates are now advancing closer to the threshold for global health impact.

## TANGIBLE PROGRESS ON A 1- OR 2-MONTH INJECTABLE

Phase III development is underway for a monthly injectable regimen containing 2 ARVs, the integrase inhibitor cabotegravir and the non-nucleoside reverse transcriptase inhibitor rilpivirine,^7^ which are intended to be used as a maintenance regimen among people who have already attained undetectable viral load on a standard 3-drug oral combination. The prospects were supported by a phase II study in which monthly or every-other-monthly injections of these 2 agents as maintenance therapy resulted in similarly high rates of viral suppression compared with patients who remained on oral treatment.^8^ Along with injectable cabotegravir's potential use in treatment, it is also now in late-stage clinical development as an every-other-monthly injection for HIV prevention.^9^ Cabotegravir is highly effective in preventing HIV acquisition in animal models,^10^ and hopes are high that it might be similarly effective in humans.

### Drawbacks of Injectable Cabotegravir and Rilpivirine

Although the potential of long-acting ARVs is clear, the current formulations of cabotegravir plus rilpivirine have several suboptimal characteristics that would likely limit their ability to achieve widespread population-level health impact, especially in the low- and middle-income countries of the world where the vast majority of people living with HIV reside. Limitations of these current investigational products include:

1. Dosing volumes are high (3 mls or more), with injections given intramuscularly in the buttocks, a relatively uncomfortable approach for patients compared with subcutaneous injection.
2. An extended tail of subtherapeutic residual levels of the drug may occur when patients stop the long-acting drugs,^11^ which could increase the risk of resistance among patients who are lost to follow-up.
3. Deliverability of injections is resource-intensive, requiring staff time and frequent patient clinic visits with a dosing frequency of every 1–2 months.
4. An oral lead-in period may be required to rule out rare hypersensitivity reactions that might arise, complicating implementation.

Further downsides specific to the rilpivirine formulation component of the treatment regimen include that it requires a cold chain, is not compatible with the commonly used tuberculosis medication rifampicin,^12^ and has a relatively weak barrier to resistance.

## NEW ARVS MAY HELP OVERCOME THESE DOWNSIDES

The good news, however, is that the cabotegravir and rilpivirine nanoemulsion combination may only be the first-generation forerunner of a much more expansive armamentarium of long-acting antiretroviral agents and delivery systems in the pipeline. One key characteristic for ARV agents to be considered candidates for long-acting systemic use is a low daily dose—the lower the better—in order for the volume of injection or size of implant to be acceptable and the cost to be minimal. Thankfully, in addition to cabotegravir and rilpivirine, there are now several other types of agents with potential to be at least as potent, and perhaps even more so. Further, these long-acting ARV candidates have resistance profiles that are highly complementary to one another. This could allow them to be combined in various ways in regimens for both treatment-naïve and treatment-experienced patients, and also reduces concern about cross-resistance emerging to treatment if some of these agents are used for prevention.

A key characteristic for ARV agents to be considered candidates for long-acting systemic use is a low daily dose.

One promising new ARV belongs to a totally new class of ARVs—the capsid inhibitor, GS-CA1, which has extremely high *in vitro* potency. A single injection of a long-acting formulation in animals produced levels for at least 10 weeks well above those necessary to inhibit viral replication.^13^ Beyond GS-CA1, there are several other promising new nucleoside/tide reverse transcriptase inhibitors (NRTIs) that may have the necessary potency for long-acting formulations, including EFdA,^14^ GS-9131,^15^ and tenofovir alafenamide fumarate (TAF).^16^ These NRTIs have the advantage that their active intracellular metabolites have very long half-lives, as they are effectively trapped within cells, thereby amplifying their levels and potency. A single oral dose of only 10 mg of EFdA in humans inhibited replication for at least 1 week,^17^ and studies in rats involving an implant suggested that greater than 6 months' release of EFdA at adequate levels may be feasible.^17^ Although EFdA is only in early-stage development, TAF is another NRTI that is already approved as an oral agent and is now being explored as an agent to be released from several different types of long-acting implants.^18^^,^^19^ And GS-9131 is an early-stage NRTI prodrug with a structure and potency somewhat similar to TAF, but with a very robust barrier to resistance and unique resistance profile.^15^ Of note, a prodrug that has the same active metabolite as GS-9131 has been described that is much more potent *in vitro* (>20 fold) and produced about fivefold greater levels of active metabolites within cells after intravenous injection in dogs compared with GS-9131.^15^ Prodrugs of cabotegravir have also been developed that, when formulated as nanoparticles, attained higher levels for a substantially longer duration in target tissues in animal studies.^20^ Substantial improvements in potency and pharmacokinetics offered by such prodrug approaches could enable longer duration of actions and/or smaller volumes of implants or injections (Table).

**TABLE.** Antiretrovirals With Long-Acting Potential: Illustrative Examples from Different Drug Classes

Drug Class	ARV Name	Development Phase	Company
Non-Nucleoside Reverse Transcriptase Inhibitor	Rilpivirine	Phase III injectable (oral agent approved)	Janssen
Integrase Inhibitor	Cabotegravir	Phase III injectable	ViiV
Nucleoside/Nucleotide Reverse Transciptase Inhibitor (NRTI)	EFdA	Phase I	Merck
	Tenofovir alafenamide fumarate (TAF)	Preclinical implant (oral agent approved)	Gilead
	GS-9131	Preclinical/Phase I	Gilead
Capsid Inhibitor	GS-CA1	Preclinical	Gilead

## BROADLY NEUTRALIZING ANTIBODIES: A POSSIBLE ALTERNATIVE TO LONG-ACTING ARVS FOR PREVENTION

In addition to small-molecule ARVs with long-acting potential, it is important to note that broadly neutralizing antibodies (bnAbs) are another type of long-acting agent that holds promise, particularly for prevention. Many highly potent bnAbs have been identified within recent years, which can block HIV viruses from multiple different clades and have been demonstrated to prevent HIV acquisition in macaques.^21^ Two large trials are now underway with a bnAb called VRC01 to attempt to demonstrate the scientific proof of principle that neutralizing antibodies can prevent infections in people.^22^^,^^23^ Although VRC01 needs to be given intravenously, which would not be practical for a prevention product, combinations of other bnAbs with expanded breadth can neutralize virtually all HIV strains at concentrations that are several orders of magnitude (about 100-fold) lower than VRC01.^24^ Further, so-called “LS” (linker substitution) mutations have been made in antibodies, which enhance neonatal Fc receptor binding and thereby greatly extend the *in vivo* half-lives of antibodies,^25^ which are already quite long. The combination of these 2 approaches, increasing potency and half-life, could potentially enable antibody-based products to be developed that could be given on a 6-monthly basis by subcutaneous injection for prevention. However, even if bnAbs do show success in human trials, for them to be a competitive alternative long-acting ARV in low-income countries would require demonstrating the feasibility of manufacturing antibodies at high scale and reasonable cost, and also of formulating them in so that they would stable in settings with weak cold-chain infrastructure.

## IMPLANTS THAT ARE EASIER TO IMPLEMENT: BIODEGRADABLE AND REFILLABLE APPROACHES

Given these significant (albeit surmountable) challenges in advancing optimized antibody-based products, it seems likely that ARVs rather than antibodies may be the first agents widely available as products in low- and middle-income countries. Along with the improved low-dose ARVs themselves, the formulation or devices through which long-acting ARVs are delivered will be a critically important component influencing effectiveness, patient acceptability, deliverability, and cost of the ultimate products. Silicone matrix implants are widely used for contraceptive implants and cost less than US$10 for generic versions of an implant that is effective for 5 years.^26^ But the daily doses required for long-acting hormonal contraception, which, after an initial burst phase, typically release doses of active agents of 50 micrograms or less per day,^27^ is about 2 orders of magnitude less than the daily amount to be released from the current long-acting nanoemulsion of cabotegravir. Thus, even if some of the new very potent, long-acting ARV candidates prove to be substantially more potent in humans than cabotegravir, it seems only to be realistic currently to imagine a duration of up to 1 year, even with an optimal formulation. For implants, this raises an implementation issue, as annual removal and replacement may not be feasible or acceptable, particularly in resource-constrained settings.

It seems likely that ARVs rather than antibodies may be the first agents widely available as products in low- and middle-income countries.

One possible approach to address this problem is to make the implants biodegradable, so that they do not require removal. While developing such biodegradable implants has long been a goal for long-acting reversible contraception, it has proved elusive due to several potential challenges, including a prolonged tail of low levels of the active agent and the possibility of dumping large amounts of the agent when the implant terminally biodegrades. However, new designs for biodegradable implants may overcome these challenges for ARVs, such as a thin-film polymer device that contains a thin biodegradable coat and a reservoir filled with an ARV (TAF, noted above, is an ARV that is being used in one investigational device).^18^ The ARV diffuses through the membrane, allowing the reservoir to gradually empty after which the outer coat biodegrades, thereby decoupling the biodegradation from the release and hopefully avoiding dumping or a prolonged tail. In addition to improved designs of biodegradable implants, another approach that might make implants more practicable for implementation is to make them refillable, so that they do not need to be implanted or removed repeatedly. One such refillable device involving nanochannels has been recently described that showed potential to release several different types of ARVs at relatively high dosages.^28^

## IMPROVED INJECTION APPROACHES: SIMPLIFYING ADMINISTRATION, REDUCING COST AND DISCOMFORT

Along with improved implants, other approaches should also be explored that may simplify delivery of injectable ARVs, such as devices that allow patients themselves and/or community health workers to more easily deliver long-acting ARVs in a manner through which pain is minimized. In this regard, easier-to-use injection devices have been developed that enable subcutaneous administration of key global health medicines such as the DMPA (depot medroxyprogesterone acetate) contraceptive injectable^29^ or oxyctocin,^30^ and microneedle-based patches are another promising approach for simplifying delivery of injections.^31^ With several candidates in the pipeline that could conceivably have low enough dosages and volumes to enable quarterly dosing for prevention and possibly even treatment, these types of simplified delivery approaches may be important to enable such injections to be implemented in a feasible, affordable, and accessible way, particularly in low-income countries. The cost of manufacturing and supplying the delivery device, while it may seem a small part of the cost for affluent countries, can be a very significant, and sometimes prohibitive, cost for low-income countries. Considering the needs of low-income countries at an early stage of product development when design decisions are being made will be critical to increase the likelihood that long-acting products that come to market will be feasible and affordable for these settings.

## COLLABORATIVE EFFORTS TO ADVANCE NEW ARVS

In recent years, public and private partners have intensified collaborative efforts to accelerate the development and introduction of optimized oral ARV regimens for low- and middle-income countries.^32^ These efforts have been motivated in part by the desire to minimize the long delays that occurred in the past between when improved ARV agents were made available in affluent countries and when they were introduced in low- and middle-income countries, such as the 7-year lag between 2006 when Atripla (efavirenz, emtricitabine, tenofovir disoproxil fumarate) was adopted as the first once-daily fixed-dose combination in the United States and when a similar generic combination was recommended as the preferred first-line regimen in the 2013 World Health Organization guidelines. Requirements for products for resource-limited settings may be more stringent than for affluent countries for characteristics such as lack of cold-chain dependence, cost of manufacturing, safety in pregnancy, and compatibility with tuberculosis medications. Therefore, developing long-acting products for these parameters, particularly for treatment which would require multiple ARVs, may necessitate especially proactive collaboration and planning, both to design and prioritize the most promising ones, as well as to ensure they are tested in a timely fashion in priority populations such as pregnant women and children.

Developing long-acting products may necessitate especially proactive collaboration and planning among public and private partners.

## CONTINUING DOWN THE ROAD TO ULTIMATE SUCCESS

The many people and organizations involved in the successful development of long-acting agents thus far deserve praise for their ingenuity, hard work, and collaboration. With several highly potent agents in the pipeline and a healthy proliferation of many promising technologies for long-acting delivery, the prospects for very long-acting ARVs for treatment and prevention have never been brighter. But to be ultimately successful, ideally with a choice of strong long-acting ARV systems, all the exemplary efforts will need to be vigorously sustained and focused on developing products well fit for implementation not only in in affluent countries but also in low-income ones. If the current promise is fulfilled, long-acting ARVs can be the keystone in finally controlling the HIV epidemic, and many of the technological and scientific advances made in the process will have application to combatting other global health scourges, multiplying the long-term impact of these efforts.

The prospects for very long-acting ARVs for treatment and prevention have never been brighter.
